# Assessment of a six gene panel for the molecular detection of circulating tumor cells in the blood of female cancer patients

**DOI:** 10.1186/1471-2407-10-666

**Published:** 2010-12-03

**Authors:** Eva Obermayr, Fatima Sanchez-Cabo, Muy-Kheng M Tea, Christian F Singer, Michael Krainer, Michael B Fischer, Jalid Sehouli, Alexander Reinthaller, Reinhard Horvat, Georg Heinze, Dan Tong, Robert Zeillinger

**Affiliations:** 1 Department of Obstetrics and Gynecology, Comprehensive Cancer Center, Medical University of Vienna, Vienna, Austria; 2 Institute for Genomics and Bioinformatics, Graz University of Technology, Graz, Austria; 3 Department of Medicine I, Comprehensive Cancer Center, Medical University of Vienna, Vienna, Austria; 4 Department of Blood Group Serology and Transfusion Medicine, Medical University of Vienna, Vienna, Austria; 5 Department of Gynecology, European Competence Center for Ovarian Cancer, Charité - University Medicine of Berlin, Berlin, Germany; 6 Clinical Institute of Pathology, Comprehensive Cancer Center, Medical University of Vienna, Vienna, Austria; 7 Section of Clinical Biometrics, Center for Medical Statistics, Informatics and Intelligent Systems, Comprehensive Cancer Center, Medical University of Vienna, Vienna, Austria; 8 Ludwig Boltzmann Gesellschaft - Cluster Translational Oncology, A-1090 Vienna, Austria

## Abstract

**Background:**

The presence of circulating tumor cells (CTC) in the peripheral blood of cancer patients has been described for various solid tumors and their clinical relevance has been shown. CTC detection based on the analysis of epithelial antigens might be hampered by the genetic heterogeneity of the primary tumor and loss of epithelial antigens. Therefore, we aimed to identify new gene markers for the PCR-based detection of CTC in female cancer patients.

**Methods:**

Gene expression of 38 cancer cell lines (breast, ovarian, cervical and endometrial) and of 10 peripheral blood mononuclear cell (PBMC) samples from healthy female donors was measured using microarray technology (Applied Biosystems). Differentially expressed genes were identified using the maxT test and the 50% one-sided trimmed maxT-test. Confirmatory RT-qPCR was performed for 380 gene targets using the AB TaqMan^® ^Low Density Arrays. Then, 93 gene targets were analyzed using the same RT-qPCR platform in tumor tissues of 126 patients with primary breast, ovarian or endometrial cancer. Finally, blood samples from 26 healthy women and from 125 patients (primary breast, ovarian, cervical, or endometrial cancer, and advanced breast cancer) were analyzed following OncoQuick enrichment and RNA pre-amplification. Likewise, *hMAM *and *EpCAM *gene expression was analyzed in the blood of breast and ovarian cancer patients. For each gene, a cut-off threshold value was set at three standard deviations from the mean expression level of the healthy controls to identify potential markers for CTC detection.

**Results:**

Six genes were over-expressed in blood samples from 81% of patients with advanced and 29% of patients with primary breast cancer. *EpCAM *gene expression was detected in 19% and 5% of patients, respectively, whereas *hMAM *gene expression was observed in the advanced group (39%) only. Multimarker analysis using the new six gene panel positively identified 44% of the cervical, 64% of the endometrial and 19% of the ovarian cancer patients.

**Conclusions:**

The panel of six genes was found superior to *EpCAM *and *hMAM *for the detection of circulating tumor cells in the blood of breast cancer, and they may serve as potential markers for CTC derived from endometrial, cervical, and ovarian cancers.

## Background

Worldwide, more than two million women are diagnosed with breast, cervical, endometrial or ovarian cancer each year. These cancers contribute to 45% of total female malignancies and approximately 880000 cancer related deaths annually [1]. Although several improvements have been made in early diagnosis during the past few decades, many patients still die of visceral metastasis, which is the main cause for tumor-related death. In these patients, the hematogenous spread of malignant cells remains undetected at the time of initial therapy. Since T. R. Ashworth first reported circulating tumor cells (CTC) in the blood of cancer patients in 1869 [2], the presence of CTC has been described for several solid tumors, such as colorectal, lung, kidney, squamous oesophageal, liver, prostate and pancreatic cancer [3]. Among cancers specific to women, the majority of CTC based research has been performed in breast cancer patients (reviewed in [3-6]), whereas few data exist for CTC in ovarian [7,8], cervical [9], and endometrial cancer [10,11] patients. Recent studies have demonstrated the prognostic role of CTC [12-14]; and the presence of tumor cells in the peripheral blood was considered to be established as an additional staging parameter [15]. Hence, many efforts have been made to develop reliable procedures for the sensitive and specific detection of CTC, either at the protein level (antibody-based cell staining) or at the mRNA level (reverse transcription PCR). While the first approach is the gold standard technique for the detection of tumor cells in the bone marrow of breast cancer patients, the latter has been proven to be more sensitive and amenable to high-throughput analysis [6]. Nevertheless, the detection of CTC is often hampered by the heterogeneity of the primary tumor and by the loss of epithelial antigens as occurs during epithelial to mesenchymal transition [3]. It has been shown that normal-like breast cancer cells characterized by aggressive behaviour and worse treatment options are not recognized by the CellSearch circulating tumor cell test (Veridex LLC, San Diego, CA), which uses EpCAM for cell isolation [16]. This test is the only diagnostic test that is currently approved by the US Food and Drug Administration for the automated detection and enumeration of circulating tumor cells [17]. *EpCAM *(epithelial cell adhesion molecule) is not a perfect marker for CTC detection due to the high variation in its gene expression between tumor subtypes and its illegitimate transcription from leukocytes [18],. Likewise, the analysis of *hMAM *(human mammaglobin A), the most widely studied marker after *CK19 *(cytokeratin 19) in breast cancer patients, gene expression identifies patients with nearly 100% specificity at the same sensitivity as *CK19 *(1 tumor cell in 10^6 ^peripheral blood mononuclear cells) [19,20]. Nevertheless, mammaglobin A gene expression is highly variable in female cancers and is detected in the blood of approximately 10 to 30% of breast cancer patients [21]. Hence, there is a high scientific and clinical need for the identification of new markers for the detection of CTC.

In this study, we aimed to identify new gene markers for the RT-qPCR based detection of CTC in the blood of female cancer patients following a step-down strategy utilizing a whole genome analysis with oligonucleotide microarrays (Applied Biosystems) and TaqMan^® ^Low Density Array (TLDA) based RT-qPCR using microfluidic technology. Based on the results of these experiments, a panel of six candidate gene markers was selected for future routine diagnosis of circulating tumor cells.

## Methods

### Experimental plan

**Figure 1:**
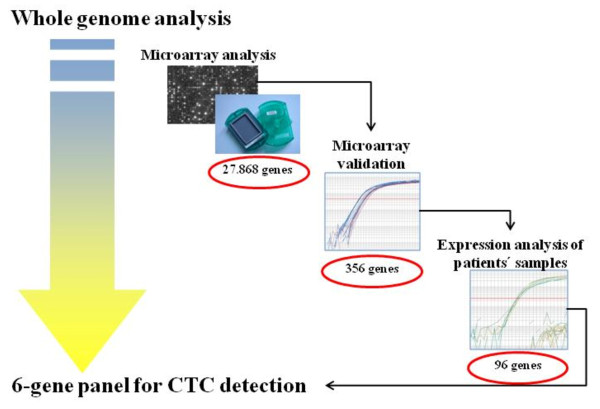
**Graphical scheme of the experimental plan**. Following a step down strategy, six genes from initially 27.686 genes were identified as new gene markers for the RT-qPCR based detection of circulating tumor cells (CTC). In microarray analysis, we compared expression profiles of PBMC isolated by Ficoll gradient centrifugation from healthy individuals and various established cancer cell lines. In microarray validation, we compared expression profiles of PBMC isolated by Oncoquick from healthy individuals and cell lines. cDNA was amplified according to a published protocol [25]. For the experimental analysis of patients samples, we used Oncoquick only. cDNA was amplified using the TargetAmp™1-Round aRNA Amplification Kit.

A step down strategy as depicted in Figure 1 was followed to identify gene markers for the detection of CTC. In the first step, microarray analysis of tumor cell lines and peripheral blood mononuclear cells (PBMC) from healthy female donors was performed. Second, the expression levels of a subset of all differentially expressed genes and of further known or supposed CTC markers were verified with RT-qPCR using the AB TaqMan^® ^Low Density Array (TLDA) platform. In the third step, genes with absent or very low expression levels in healthy PBMC were selected for the analysis of blood samples taken from patients before initial surgery of the primary tumor using the same RT-qPCR platform. As the number of circulating tumor cells was suspected to be low, a RNA pre-amplification step was performed. Again, a healthy control group was analysed. The aim of the third step was to identify new gene markers for the RT-qPCR based detection of CTC.

### Ethical considerations

The study was approved by the Ethics Committee from the Medical University of Vienna, Austria (reference numbers 366/2003 and 260/2003) and by the Institutional Review Board of the Charité Hospital. All peripheral blood and tumor tissue samples were collected with the patients' written consent.

### Cell culture

Overall, 10 breast cancer cell lines (MCF-7, T-47 D, MDA-MB-231, Hs 578T, MDA-MB-435 S, MDA-MB-453, BT-474, SK-BR-3, ZR-75-1, BT-549), 10 ovarian cancer cell lines (A2780, Caov-3, ES-2, NIHOVCAR-3, SK-OV-3, TOV-21G, TOV-112 D, OV-90, OV-MZ-01a, OV-MZ-6), 9 cervical cancer cell lines (HeLa, SW756, GH354, Ca Ski, C-4 I, C-33 A, HT-3, ME-180, SiHa), and 9 endometrial cancer cell lines (KLE, RL95-2, AN3 CA, HEC-1-B, Ishikawa, Colo 684, HEC-50-B, EN, EJ) were cultivated according to the recommended protocols and harvested on at least three consecutive days. If commercially available, the cell lines were purchased from the American Type Culture Collection (ATCC, http://www.atcc.org) or from the European Collection of Cell Cultures (ECACC, http://www.ecacc.org.uk).

### Peripheral blood and tumor tissues

From 2001 to 2006 peripheral blood (PB) samples were collected from 567 patients with malign gynecological diseases at the Department of Obstetrics and Gynecology and at the Department of Medicine I, Division of Oncology (all located at the MUW Medical University of Vienna, A). Patients with tumors of low malignant potential (i.e. borderline tumor of the ovaries), with concomitant or previous malignant tumors other than from the breast, the ovaries or the uterus, transplanted patients, and pregnant patients were excluded. Finally, we included one blood sample from 94 patients (21 breast, 23 ovarian, and each 25 cervical and endometrial cancer patients) taken before the initial treatment (excision of the primary tumor or administration of systemic neoadjuvant chemotherapy). Additionally, we analyzed one blood sample from 31 patients with recurrent breast cancer and distant metastasis.

PB taken from 58 healthy female volunteers at the MUW Department of Blood Group Serology and Transfusion Medicine, the MUW Department of Obstetrics and Gynecology and ViennaLab Diagnostics GmbH (Vienna, A) served as negative controls. All PB samples were collected in EDTA tubes and processed within 2 hours after venipuncture. The patient characteristics are given in Table 1.

**Table 1:** Base line characteristics of patients included into the RT-qPCR analysis of peripheral blood.

	Venipuncture	
	**A**	**B**

Total number of patients	94	31

Breast cancer		

Number	21	31

Age (yrs)		

median	54	50

range	35-78	25-75

Histology		

IDC	100.0%	64.5%

ILC	0	12.9%

Others/unknown	0	22.6%

TNM Stage *		

I	38.1%	3.2%

II	33.3%	48.4%

III	23.8%	19.4%

IV	4.8%	3.2%

Unknown	0	22.6%

Endometrial cancer		

Number	25	0

Age (yrs)		

median	64	

range	30-83	

Histology		

Endometrioid	100.0%	

FIGO Stage		

I	60.0%	

II	8.0%	

III	28.0%	

IV	4.0%	

Cervical Cancer		

Number	25	0

Age (yrs)		

median	48	

range	29-78	

Histology		

Non-keratinizing	48.0%	

Keratinizing	40.0%	

Others/unknown	12.0%	

FIGO Stage		

I	8.0%	

II	44.0%	

III	28.0%	

IV	20.0%	

Ovarian cancer		

Number	23	0

Age (yrs)		

median	53	

range	37-78	

Histology		

Serous	72.7%	

Mucinous	12.1%	

Others/unknown	15.1%	

FIGO Stage		

I	10.0%	

II	10.0%	

III	65.0%	

IV	15.0%	

Peripheral blood was taken from 94 patients before initial treatment of the primary tumor (A). From 31 recurrent breast cancer patients (B) the blood was taken during disease progression (* TNM stage refers to the primary tumor).

In the same time period, fresh frozen tissue samples from patients with breast, ovarian, endometrial or cervical carcinoma were kindly provided by the MUW Department of Gynecopathology, Clinical Institute for Pathology. Additional ovarian cancer tissues were collected by the Department of Obstetrics and Gynecology at the Charité-Universitätsmedizin Berlin (TOC = Tumorbank Ovarian cancer) (D). All tissue samples were stored in liquid nitrogen prior to homogenization. The study inclusion criteria were the same as for blood samples; furthermore, recurrent patients and tissue samples taken after neoadjuvant chemotherapy were excluded. From a total of about 340 collected tumor tissues 50, 51 and 25 patients with primary breast, ovarian or endometrial cancer, respectively were enrolled in the study.

The patient characteristics are summarized in Additional file 1.

### Cell spiking

For sensitivity assays, a defined number of T-47 D breast cancer cells were added to each 15 ml pre-cooled PB sample obtained from a healthy female donor and provided by the Austrian National Red Cross Society. An unspiked blood sample from the same donor served as a negative control. Each blood sample was spiked in duplicate. Samples were enriched by OncoQuick (Greiner Bio-One, Frickenhausen, D) per the manufacturer's instructions, resuspended in RLT-buffer (Qiagen RNA Isolation Kit), and the corresponding lysates pooled to compensate for varying recovery rates of the enrichment procedure. 1/6 of the extracted total RNA (Qiagen RNA Isolation Kit) was pre-amplified in triplicate reactions employing the TargetAmp™1-Round aRNA Amplification Kit (Epicentre, Madison WI, USA) according to manufacturer instructions. The pre-amplified RNA was converted into cDNA with M-MLV Reverse Transcriptase, RNase H Minus (Promega, Madison WI, USA) and random hexamers as primers. To assess the sensitivity of the TLDA platform to detect circulating tumor cells, RT-qPCR was performed using the TLDA format 96a as described below.

### Sample processing

For the microarray-based gene expression studies, PBMC were isolated from 50 ml healthy female blood by a density gradient using Ficoll-Paque™Plus (GE Healthcare Bio-Sciences AB, Uppsala, S) per the standard procedure. For gene expression analysis with RT-qPCR, which required an enhanced depletion of leukocytes than is provided by Ficoll, mononuclear cells from 15-25 ml PB taken from healthy females and patients were enriched using OncoQuick^® ^tubes (Greiner Bio-One, Frickenhausen, D) according to the manufacturer's instructions.

100 mg of fresh frozen tumor tissue was pulverized for 2 min at 2000 rpm using a microdismembrator (B. Braun Biotech., Melsungen, D) and further homogenized in lysis solution by intense vortexing.

### RNA extraction

Total RNA was extracted with two commercially available kits depending on the amount of cells in the starting material: First, the Total RNA Isolation Mini Kit (Agilent Technologies, Waldbronn, D) was used for RNA extraction from cultivated tumor cells, from homogenized tumor tissue and from PBMC enriched by Ficoll-Paque™Plus density gradient centrifugation. Total RNA samples were spectrophotometrically quantified and examined for residual genomic DNA by PCR employing primers which span exon 9 of the breast cancer 2, early onset gene *BRCA2 *(sense primer: 5'-ATA ACT GAA ATC ACC AAA AGT G-3'; antisense primer: 5'-CTG TAG TTC AAC TAA ACA GAG G-3'). Residual genomic DNA was digested by DNase I. Finally, quality assessment of the cell line- and PBMC-RNA was performed with the RNA 6000 Nano LabChip Kit run on the 2100 Bioanalyzer (Agilent Technologies, Waldbronn, D) and of RNA samples isolated from tumor tissues with denaturing agarose gel electrophoresis. The total RNAs extracted from at least three consecutive cell line harvests were combined to compensate for differences in expression that may result from varying culture conditions. Each of the RNA pools and the RNA samples extracted from healthy PBMC were precipitated to reach a minimal final concentration of 1.5 μg/μl. Second, the RNeasy Micro Kit (Qiagen, Hilden, D) was used for RNA extraction from cells enriched by Oncoquick^® ^gradient. Because we expected low RNA yields, we restrained from losing further material by assessing the RNA quality or quantity in these samples.

### Expression profiling using Human Genome Microarrays

A total of 48 Human Genome Survey Microarrays Hs.v1 (Applied Biosystems, Foster City CA, USA) were performed to compare the gene expression of 38 tumor cell lines and 10 healthy control samples at GeneSys Laboratories GmbH (Muenster, D) under standard conditions using kits, reagents and the chemiluminescent microarray analyzer 1700 from AB. In brief, 20 μg of total RNA was used to prepare digoxigenin-labeled cDNA, which developed a chemiluminescent signal after hybridizing to the 60-mer oligonucleotide probes spotted onto the microarray platform. Primary analysis and quality control were performed using the AB Navigator Software Version 1.0.0.3. After background correction, data were normalized using the ABI 1700 Chemiluminescent Analyzer first by feature, then by spatial effects in the slide. Finally a global normalization per slide was performed. AB provides the normalized data in the column assay normalized signal (ANS). The log base 2 ANS was considered for further analysis. Microarray expression measurements with a flag of greater than 5000 indicating a low quality spot were filtered out. These measurements generally correlate with spots that have a signal to noise ratio smaller than or equal to 3. Since we are interested in genes that are not expressed in healthy controls, only those gene probes with an average ANS in the control samples that was smaller than 1.5 were subjected to statistical analysis. We performed two statistical tests in parallel to identify differentially expressed genes. For the maxT test from the multtest Bioconductor package [22], genes were considered differentially expressed if they contained a corrected p-value ≤ 0.05 [23] Additionally we used a 50% one-sided trimmed maxT-test [24] with a familywise error rate of 0.05 and 1000 permutations. This test resembles the ordinary maxT test but replaces the t-statistic with a trimmed t-statistic in both the original and the permuted data. For each gene of the original and each permuted data set, the trimmed t-statistic is computed from only those data values, which are greater than the group medians. In contrast to the maxT test, the 50% one-sided trimmed maxT-test can identify genes, which are over-expressed in only a subgroup of the tumor cell lines.

Finally, 356 genes with a mean difference between tumor cell lines compared to the healthy control samples of greater than 10 were selected for confirmatory gene expression profiling by RT-qPCR using the AB TaqMan^® ^Low Density Array (TLDA) platform. Additionally, the selected 356 genes were supplemented with 15 known or supposed markers for CTC detection.

### Verification of microarray results with RT-qPCR

The expression levels of the 356 genes selected from the microarray analyses and of the 15 known or supposed CTC markers were verified with RT-qPCR in a subset of each five breast, ovarian and endometrial cancer cell lines and in blood samples from 19 healthy females. RT-qPCR was performed on the AB 7900HT Fast Real-time PCR System per manufacturer instructions using the TLDA format 384 for the analysis of 380 gene targets in single reactions and of one mandatory endogenous control gene (glyceraldehyde-3-phosphate dehydrogenase [*GAPDH*]) in a quadruplicate reaction. The 380 gene targets consisted of the 3 additional TaqMan^® ^Endogenous Controls (beta-2-microglobulin [*B2M*], TAT-box binding protein [*TBP*], and phosphoglyceratekinase 1 [*PGK*]) and 377 TaqMan^® ^Gene Expression Assays specific for the 15 known or supposed CTC marker and specific for the previously selected differentially expressed genes according to a mapping of microarray probe IDs to assay IDs provided by AB. The RNA extracted from tumor cell lines was converted into cDNA with M-MLV Reverse Transcriptase, RNase H Minus (Promega, Madison WI, USA) and random hexamers as primers. Blood mononuclear cells were enriched with Oncoquick density gradient centrifugation. Then, the extracted RNA was amplified following a modified version of a protocol published by Klein et al. [25]. In short, the RNA was first converted into cDNA with M-MLV Reverse Transcriptase, RNase H Minus (Promega, Madison WI, USA) and random primers containing a 5'-oligo-dC flanking region (5'-[CCC]_5 _TGC AGG N_6_-3'; VBC Genomics, Vienna, A). After generating a 3'-oligo-dG flanking region, the flanked cDNA was primed with CP2 (5'-TCA GAA TTC ATG [CCC]_5_-3'; VBC Genomics) and amplified with Super Taq (HT Biotechnology Ltd., Cambridge, GB). The TLDA were loaded with the sample-specific PCR mix containing the template cDNA as recommended by the manufacturer (2 ng per well). Raw data were analyzed with the AB 7900 Sequence Detection Software version 2.2.2 using automatic baseline correction and a manual cycle threshold (C_t_) setting. Resulting C_t _data was exported for further analysis. To downsize the number of potential candidate genes from initially more than 27.000 genes to about 100 genes, all genes with expression levels beyond the RT-qPCR detection limit (i.e. C_t _50) in the healthy control samples were excluded. The remaining genes were sorted in descending order by their average C_t _value obtained from the 15 tumor cell lines. The first 93 genes were selected for RT-qPCR analysis of blood and tissue samples taken from tumor patients using the TLDA 96a format. Additionally, three genes (*B2M*, *GAPDH *and *PGK*) were selected as internal reference genes.

### Gene expression analysis of tumor tissue samples

The expression of the previously selected 93 genes was measured in tumor tissue samples from patients with primary breast (N = 50), ovarian (N = 51) and endometrial cancer (N = 25) with RT-qPCR using the TLDA 96a format to verify their use as candidate markers for the detection of CTC in the blood of cancer patients. RNA was converted into cDNA by Omniscript Reverse Transcriptase (Quiagen, Hilden, D) using an oligo-dT-flanked primer. Loading the microfluidic cards, RT-qPCR amplification, and raw data analysis were performed as described in the last preceding section. All samples were analyzed in duplicates.

### Gene expression analysis of patients' blood samples

The expression of the same 93 genes was evaluated in blood samples from healthy female volunteers (N = 26) and in peripheral blood samples from patients with breast (N = 52), ovarian (N = 23), cervical and endometrial cancer (25 patients each), using the TLDA 96a RT-qPCR platform. After cell enrichment with OncoQuick density gradient centrifugation 1/6 of the total RNA was amplified employing the TargetAmp™1-Round aRNA Amplification Kit (Epicentre, Madison WI, USA) per manufacturer instructions. The amplified RNA was converted into cDNA with M-MLV Reverse Transcriptase, RNase H Minus (Promega, Madison WI, USA) and random hexamers as primers. Loading of the microfluidic cards, RT-qPCR amplification, and raw data analysis were performed as described in the microarray verification section. All samples were analyzed in duplicate. The mean of the resulting duplicate C_t _values was used as a quantitative value. If only one of the duplicates was positive (i.e. C_t_ < 50), the positive C_t _value was taken. Low-level expression of many genes in the peripheral blood of the healthy control group decreased the overall specificity of the assay and required the introduction of a cut-off threshold value to separate the cancer patient group from the healthy control group:

As proposed by Mikhitarian et al. [26], a threshold value T_X _for each gene X was set to three standard deviations from the mean dC_tX _value in the control group. dC_tX _values were calculated by normalizing the average expression of gene X to the average expression of the endogenous control gene *GAPDH*. If only one healthy control sample revealed detectable gene expression, the one dC_tX _was taken as cut-off threshold value. A tumor patient was considered positive for the molecular analysis of gene × if dC_tX _was below the defined threshold value T_X_.

Additionally, *hMAM*- and *EpCAM*-specific RT-qPCR was performed for the same set of breast and ovarian cancer blood samples and for healthy female control samples after cell enrichment and RNA pre-amplification as described above using individual AB TaqMan^® ^Pre-Developed Assay Reagents (Hs00267190_m1 and Hs00158980_m1).

## Results

### RNA quality assessment

Prior to microarray hybridization and RT-qPCR analysis, the RNA extracted from the tumor cell lines and the healthy PBMC was checked for quality with the RNA 6000 Nano LabChip Kit run on the Agilent 2100 Bioanalyzer. As a result, 85% of the RNA samples were of very good RNA quality (RIN≥8), 60% of which were considered to have an excellent quality (RIN≥9).

### Differentially expressed genes in tumor cell lines compared to healthy PBMC

We compared the gene expression profile of 38 established cancer cell lines to those of PBMC taken from 10 healthy donors to identify genes that were not expressed or expressed at very low level in the peripheral blood of healthy females but appeared very highly expressed in the cancer cell lines. From the 18151 (54.8%) genes with an average ANS < 1.5 in the healthy control samples maxT-test identified 457, 534, 526, and 503 genes differentially expressed for the breast, cervical, endometrial, and ovarian cancer cell lines, respectively. These genes comprised 54, 81, 63, and 60 genes with cancer-type specific expression for the respective cancer cell lines. Additionally, the 50% one-sided trimmed maxT-test identified further 25, 27, 20 and 29 genes, which were differentially expressed in the breast, cervical, endometrial and ovarian cancer cell lines compared to the healthy controls.

Finally, 356 differentially expressed genes were chosen for confirmatory gene expression profiling with RT-qPCR using the TLDA 384 format (microarray data are provided in Additional file 2). This consisted of 337 genes identified by the maxT-test, 19 by the 50% one-sided trimmed maxT-test only, and the 4 genes: *EFEMP1*, *EPS8L1*, *CRYZL1 *and *PCDHG *represented with more than one TaqMan^® ^Assay. Additionally we decided to analyze nine markers of well-known tumor specificity (*ERBB2, ESR1, PGR, PLAT, SCGB2A1, SCGB2A2, SERPINE1, SERPINE2 *and *TFF1*) and six candidate markers for CTC detection that were previously identified by our research group (*COL3A1*, *GHR*, *CALB1*, *LPHN1*, *FN1 *and *EDNRA*) [27].

### Verification of microarray results with RT-qPCR

146 genes of the TLDA 384 gene set were identified as potential markers for the detection of CTC in the blood of cancer patients with expression levels below the detection limit of RT-qPCR (i.e. C_t _50) in the healthy control group. The genes were sorted in descending order by their average C_t _value obtained from the 15 tumor cell lines, and the first 93 genes were selected for further gene expression analysis of patients' samples using the TLDA 96a format (see Additional file 3). None of the 15 known or supposed markers for CTC detection was considered for further investigations either due to detectable expression levels (*ERBB2*, *ESR1*, *SERPINE1*, *SERPINE2 *and *FN1*) in healthy controls or due to inadequate gene expression in the tumor cell lines.

### Sensitivity

**Figure 2:**
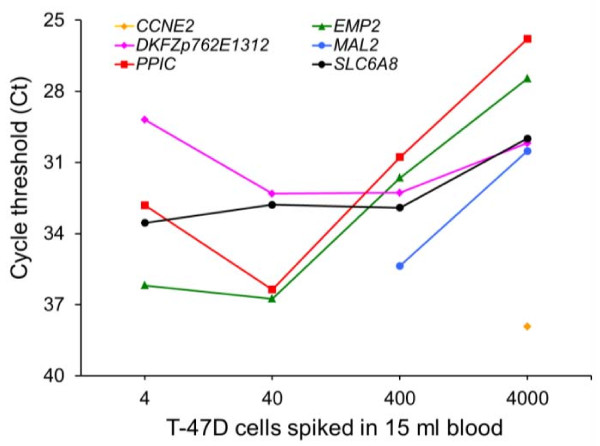
**Sensitivity of RT-qPCR using TLDA platform**. Expression levels of 93 candidate genes were analyzed using cDNA generated from total RNA isolated from peripheral blood samples from a healthy female donor and the same blood spiked with 4, 40 and 400 T47-D tumor cells after cell enrichment. RNA was pre-amplified using the TargetAmp™1-Round aRNA Amplification Kit. Average C_t _values obtained from RT-qPCR amplification of *CCNE2*, *DKFZp762E1312*, *EMP2*, *MAL2*, *PPIC*, and *SLC6A8 *transcripts using the TLDA format are shown. *MAL2 *and *CCNE2 *gene expression was below the detection limit of RT-qPCR in the unspiked blood. The detection sensitivity of the respective marker gene was estimated to be 40 and 400 tumor cells per 15 ml whole blood.

To assess the applicability of the TLDA platform for the RT-qPCR based detection of circulating tumor cells, the expression levels of the specified 93 genes were measured in healthy female blood samples spiked with T-47 D breast cancer cells. *CCNE2 *and *MAL2 *transcripts were detected in blood samples spiked with at least 26 and 3 tumor cells per ml blood, respectively (Figure 2), but they were not detected in the unspiked blood. Although background expression of *EMP2*, *PPIC, DKFZp762E1312*, and *SLC6A8 *was detected in the unspiked blood, increasing expression levels of the respective genes were observed when tumor cells had been added to the blood, with a detection limit of 3 (*EMP2*, *PPIC*) and 26 tumor cells per ml of blood (*DKFZp762E1312*, *SLC6A8*). Furthermore, the spiking experiments revealed that RT-qPCR might be less sensitive using the TLDA platform than using conventional PCR tubes, because linear amplification patterns distinguishing each 10-fold dilution were only observed with C_t _values smaller than 35 (data not shown).

### Gene expression in tumor tissues

The gene expression of the previously selected 93 genes was confirmed in tumor samples from patients with primary breast, ovarian and endometrial cancer. We observed that the house-keeping gene expression levels were lower in ovarian cancer tissues than in tumor tissues of breast and endometrial cancer patients (*GAPDH *24.2 ± 2.6, 22.2 ± 1.2, 22.7 ± 1.4 (SD) C_t_; *B2 M *22.1 ± 3.4, 18.1 ± 1.5, 17.7 ± 1.9 (SD) C_t_; *PGK *25.5 ± 2.7, 23.5 ± 1.1, 22.4 ± 3.0 (SD) C_t _in the respective tumor patients). Two of the 93 genes were found to be tumor-site specific: *PLEKHC1 *(pleckstrin homology domain containing, family C [with FERM domain] member 1) and *SGCB *(sarcoglycan beta) transcripts were detected only in ovarian cancer patients (see Additional file 4), although they were also detected in cancer cell lines of breast and endometrial origin either. Interestingly, expression of the selected 93 genes was detected in more ovarian cancer patients than in breast and endometrial cancer patients (median percentage of positive patients in the respective tumor groups was 78.4%, 64.0% and 32.0%).

### Gene markers for CTC detection

The expression of the previously selected 93 genes was evaluated in blood samples from cancer patients, to identify the most promising markers for CTC detection. At primary diagnosis, each 17 (68.0%) cervical and endometrial cancer, 6 (26.1%) ovarian cancer and 8 (38.1%) breast cancer patients over-expressed at least one out of the 93 potential candidate genes at levels above the defined threshold. At the time-point of disease recurrence, 27 (87.1%) breast cancer patients were positive for at least one gene. Of the 93 candidate genes, 40 were able to identify patients using the defined respective thresholds. 33 of these genes were capable to identify patients with primary breast cancer, and this number was reduced to 15 for patients with advanced disease stage. 14 of these genes could identify patients with cervical and endometrial cancer and four of the 40 genes identified ovarian cancer patients. The remaining 55 genes did not provide any value due to similar expression levels in both the healthy control and cancer patient groups.

**Figure 3:**
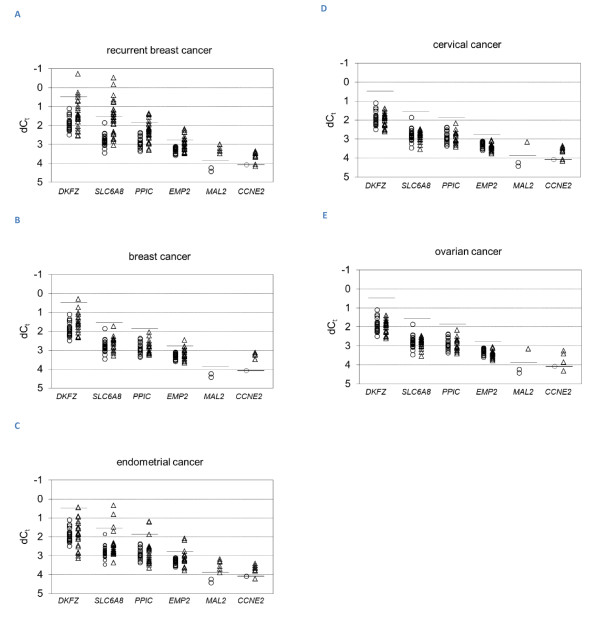
**RT-qPCR analysis of marker gene expression in peripheral blood**. Gene expression was analyzed in blood samples taken from patients (triangles) with recurrent breast cancer (A), and in blood samples taken at first diagnosis from breast (B), endometrial (C), cervical (D) and ovarian (E) cancer patients. Blood from healthy females (circles) served as a control group. Mononuclear cells were enriched with the Oncoquick density gradient. RT-qPCR was performed following a RNA pre-amplification step. Average C_t _values obtained from duplicates were normalized to *GAPDH *gene expression. Cut-off threshold values calculated from the mean average normalized gene expression in healthy female blood as indicated by horizontal lines for the respective gene markers (*DKFZp762E1312 *1.39, *SLC6A8 *2.92, *PPIC *3.61, *EMP2 *6.84, *MAL2 *14.61, *CCNE2 *16.83).

The purpose of this study was to identify a panel of genes for future multi-marker RT-qPCR based analysis to increase the sensitivity to detect circulating tumor cells. For this purpose, we selected those genes, which were over-expressed in more than 10% of the patients with recurrent breast cancer, since circulating tumor cells are more likely in advanced disease. According to this criterion, six genes (*CCNE2*, *DKFZp762E1312*, *EMP2*, *MAL2*, *PPIC *and *SLC6A8*) were chosen for a RT-qPCR marker panel. Using this panel 81% of the breast cancer patients with recurrence and 29% of the breast cancer patients at initial diagnosis were positive for at least one gene. In the cervical, endometrial and ovarian cancer groups, the percentage of positive patients was found to be 44%, 64% and 19%, respectively (see Table 2 and Figure 3).

**Table 2:** Marker gene expression in peripheral blood

	Positive blood samples (%)								
**Patients**	**Panel**	**CCNE2**	**MAL2**	**EMP2**	**SLC6A8**	**DKFZ**	**PPIC**	**hMAM**	**EpCAM**

rec. BC (N = 31)	80.6	32.3	19.4	32.3	45.2	25.8	19.4	38.7	19.4

BC (N = 21)	28.6	23.8	0	4.8	0	4.8	0	0	5.0

OC (N = 23)	19.0	13.0	4.3	0	0	0	0	0	0

EC (N = 25)	64.0	36.0	20.0	12.0	12.0	8.0	8.0	0	0

CC (N = 25)	44.0	40.0	4.0	4.0	4.0	4.0	0	0	0

Healthy (N = 26)	0	0	0	0	0	0	0	0	0

The percentage of patients with RT-qPCR positive blood samples is shown. RT-qPCR positivity was defined as gene expression beyond the cut-off threshold, which was set for each gene marker at three standard deviations from the mean expression in healthy control blood samples. Positivity in percentage shown for the "panel" (*CCNE2*, *DKFZp762E1312*, *EMP2*, *MAL2*, *PPIC *and *SLC6A8*) is defined as positivity for at least one of the markers.

(BC: breast cancer, rec. BC: recurrent breast cancer, OC: ovarian cancer, EC: endometrial cancer, CC: cervical cancer, ND: not done)

Additionally, *hMAM*-specific RT-qPCR performed for the same set of breast and ovarian cancer blood samples confirmed the tissue specific expression of mammaglobin A. Transcripts were only detected in recurrent breast cancer patients with an incidence of 38.7%, but neither in primary breast cancer patients, ovarian cancer patients, nor in the healthy controls. Likewise, *EpCAM *gene over-expression was detected in the blood of neither ovarian cancer patients nor healthy females. In the blood of breast cancer patients, we found *EpCAM *over-expression in 5.0% of the patients at primary diagnosis and in 19.4% of the patients with clinical evidence of disease recurrence (see Table 2).

## Discussion

Using a stepwise approach combining genome-wide expression profiling and TaqMan^® ^based RT-qPCR we identified six genes (*CCNE2*, *DKFZp762E1312*, *EMP2*, *MAL2*, *PPIC*, and *SLC6A8*) as potential markers for the detection of circulating tumor cells in the peripheral blood of patients with breast cancer and gynecological malignancies. Although each of these genes is implicated in cancer, they have not previously been specified for the detection of circulating tumor cells in cancer patients.

Initial screening of candidate gene markers for CTC detection was performed using a microarray-based gene expression analysis of human cancer cell lines and mononuclear blood cells obtained from healthy females. After verification of the microarray results, a set of 93 gene markers was selected for the RT-qPCR analysis of blood samples from healthy females and from patients with breast, ovarian, endometrial, and cervical cancer.

Due to background gene expression in the healthy blood samples, a rigorous cut-off threshold value was introduced to separate the patients from the healthy controls. We assumed that the over-expression of at least one gene marker in relation to the defined threshold value indicated the presence of circulating tumor cells. As patients with recurrent breast cancer are most likely to harbor circulating tumor cells in their blood, their blood samples were chosen to identify new gene markers for CTC detection. A panel of six genes: *CCNE2*, *DKFZp762E1312*, *EMP2*, *MAL2*, *PPIC*, and *SLC6A8 *that were over-expressed in the blood of 81% of patients with recurrent breast cancer was then chosen as gene markers for the molecular detection of CTC. In contrast, at initial diagnosis using the six gene panel only 29% of the breast cancer patients were RT-qPCR positive. In addition, the new gene panel identified patients with other female cancers (i.e. cervical, endometrial and ovarian cancer).

In tumor cell spiking experiments the sensitivity of the applied RT-qPCR was estimated to be 3 to 26 tumor cells per ml whole blood; similar sensitivities are reported for RT-qPCR- and immuno-mediated detection (reviewed by Gervasoni et al. [28]). However, we found out that TaqMan^® ^Low Density Arrays are typically not the method of choice for the detection of rare template molecules.

In the present study, all blood samples were taken before removal of the tumor masses. To estimate whether the six gene panel is useful to detect minimal residual disease, further experiments should include blood samples from cancer patients taken after the excision of the primary tumor. Although we have already analysed several blood samples taken from breast cancer patients with no evidence of disease six months after completion of their adjuvant chemotherapy, the follow-up time is yet too short to make any conclusions concerning the patient outcome.

There are further limitations that need to be acknowledged and addressed regarding the experimental design of the present study. First, when we evaluated various approaches for the enrichment of circulating tumor cells in in the course of the project, we found out that Oncoquick may insufficiently recover spiked tumor cells, in particular when only a few tumor cells were added to the blood (i.e. ≤ 20 tumor cells per 15 ml blood) [29]. For this reason, false-negative RT-qPCR results are likely to occur for cancer patients with low CTC counts. Second, the density of the tumor cells depends on their differentiation state. Therefore, undifferentiated tumor cells having a higher density might pass through the Oncoquick density gradient. Finally, we cannot exclude false-positive cases due to non-malignant epithelial cells, which may contaminate the blood samples during venipuncture and which express the targeted transcripts. Nevertheless, we decided in favour of the Oncoquick density gradient, because it dramatically reduced background gene expression of the selected targets in healthy PBMC samples. To enhance the sensitivity and specificity of the approach, future experiments should primarily aim at improving the recovery rate of the tumour cell enrichment. Further evaluation of the six CTC markers should be done without RNA pre-amplification and using the conventional PCR tube format instead of TaqMan^® ^Low Density Array format.

Despite these limitations, we suggest that the RT-qPCR based analysis of *CCNE2*, *DKFZp762E1312*, *EMP2*, *MAL2*, *PPIC*, and *SLC6A8 *gene expression in the blood of patients with breast cancer or gynecologic malignancies is useful for the detection of circulating tumor cells, alone or combined with other markers such as *hMAM *or *EpCAM*. Interestingly, the *DKFZp762E1312*, *EMP2*, *PPIC*, and *SLC6A8 *transcripts, but not *CCNE2 *and *MAL2 *transcripts were detected in the blood of healthy females. Therefore, we suppose that the detection of *CCNE2 *and *MAL2 *transcripts in the blood of cancer patients is indicative for CTC presence (which had not been verified by immunocytochemistry). However, the observed increase of *CCNE2 *mRNA levels in the diseased group compared to the healthy control group, which are reported to be undetectable in normal quiescent cells arrested in G_0 _[30], is in conflict with the supposed non-proliferative nature of circulating tumor cells [31]. Interestingly, both *CCNE2 *and *MAL2 *are located on chromosome 8q, a region which is frequently increased in copy number in breast [32] and other cancer types [32,33], and one of the most important target genes affected by gains and amplifications of 8q is the *MYC *oncogene.

The frequency of *hMAM *gene expression in the blood of breast cancer patients is in line with the frequencies reported by Roncella et al. [20]. 10 of the 12 *hMAM *positive blood samples (83%) were also positive when analyzed using the six gene panel, and 52% of the recurrent breast cancer blood samples were solely identified by *CCNE2*, *DKFZp762E1312*, *EMP2*, *MAL2*, *PPIC*, or *SLC6A8*. Similarly, the six gene panel identified all of the *EpCAM *positive blood samples.

## Conclusions

In this study, we identified new gene markers for the assessment of circulating tumor cells. We have shown that the RT-qPCR-based multi-marker analysis using the six genes: *CCNE2*, *DKFZp762E1312*, *EMP2*, *MAL2*, *PPIC*, or *SLC6A8 *more than doubled the number of positive patients with recurrent breast cancer compared to the analysis of *hMAM *or *EpCAM *gene expression alone. Therefore, we suggest that the significantly higher expression of these six genes in the peripheral blood indicates the presence of circulating tumor cells. This multi marker analysis may provide a tool for clinical monitoring and treatment control of breast cancer and of gynecological malignancies. Eventually it may also be useful for the early detection.

## Competing interests

ZR, having ZR, DT and EO as inventors, filed a patent application based upon this manuscript.

## Authors' contributions

EO performed and supervised sample processing, carried out the RT-qPCR analysis and data evaluation, and drafted the manuscript. FSC and GH performed statistical analysis of microarray data. CFS, MKT, AR, MK, MBF, RH and JH coordinated the collection of patients' blood and tissue samples. DT and RZ designed the study and contributed to data interpretation. RZ served as mentor for the entire project. All authors read and approved the final manuscript.

## Pre-publication history

The pre-publication history for this paper can be accessed here:

http://www.biomedcentral.com/1471-2407/10/666/prepub

## Supplementary Material

Additional file 1 **Base line characteristics of patients included into the RT-qPCR analysis of tumor tissue**. Click here for file

Additional file 2 **Microarray data of 356 differentially expressed genes**. Click here for file

Additional file 3 **Gene identifiers of the TLDA 96a platform**. 93 genes were selected as CTC candidate genes for the RT-qPCR analysis of blood and tumor tissue samples from cancer patients. Additionally, three house-keeping genes (*B2M*, *GAPDH*, and *PGK1*) were chosen as an internal reference. Click here for file

Additional file 4 **Gene expression in tumor tissues**. The percentage of breast, endometrial and ovarian cancer patients with gene expression detected by RT-qPCR is shown for each of the 93 candidate genes and for the three internal reference genes (*B2M*, *GAPDH*, and *PGK1*). Click here for file
